# Hypoxic conditions increase hypoxia-inducible transcription factor 2α and enhance chondrogenesis in stem cells from the infrapatellar fat pad of osteoarthritis patients

**DOI:** 10.1186/ar2211

**Published:** 2007-05-30

**Authors:** Wasim S Khan, Adetola B Adesida, Timothy E Hardingham

**Affiliations:** 1 UK Centre for Tissue Engineering and Wellcome Trust Centre for Cell Matrix Research, Faculty of Life Sciences, Michael Smith Building, University of Manchester, Oxford Road, Manchester M13 9PT, UK

## Abstract

Stem cells derived from the infrapatellar fat pad (IPFP) are a potential source of stem cells for the repair of articular cartilage defects. Hypoxia has been shown to improve chondrogenesis in adult stem cells. In this study we investigated the effects of hypoxia on gene expression changes and chondrogenesis in stem cells from the IPFP removed from elderly patients with osteoarthritis at total knee replacement. Adherent colony-forming cells were isolated and cultured from the IPFP from total knee replacement. The cells at passage 2 were characterised for stem cell surface epitopes, and then cultured for 14 days as cell aggregates in chondrogenic medium under normoxic (20% oxygen) or hypoxic (5% oxygen) conditions. Gene expression analysis, DNA and glycosoaminoglycan assays and immunohistochemical staining were determined to assess chondrogenesis. IPFP-derived adherent colony-forming cells stained strongly for markers of adult mesenchymal stem cells, including CD44, CD90 and CD105, and they were negative for the haematopoietic cell marker CD34 and for the neural and myogenic cell marker CD56. Cell aggregates of IPFP cells showed a chondrogenic response. In hypoxic conditions there was increased matrix accumulation of proteoglycan but less cell proliferation, which resulted in 3.5-fold more glycosoaminoglycan per DNA after 14 days of culture. In hypoxia there was increased expression of hypoxia-inducible transcription factor (HIF)2α and not HIF1α, and the expression of key transcription factors SOX5, SOX6 and SOX9, and that of aggrecan, versican and collagens II, IX, X and XI, was also increased. These results show that cells with stem cell characteristics were isolated from the IPFP of elderly patients with osteoarthritis and that their response to chondrogenic culture was enhanced by lowered oxygen tension, which upregulated HIF2α and increased the synthesis and assembly of matrix during chondrogenesis. This has important implications for tissue engineering applications of cells derived from the IPFP.

## Introduction

Cartilage is frequently damaged by trauma and in disease but shows only a limited capacity for repair. Most focal cartilage lesions, left untreated, progress to more extensive lesions and in the long term these require joint arthroplasty. Autologous chondrocytes harvested from low-weight-bearing areas of articular cartilage are being used for the repair of focal hyaline cartilage defects [1]. Although short-term clinical results have been good, evidence suggests some incidence of progressive degenerative changes in the joint [2]. This procedure is also accompanied by donor site morbidity, and the limited amount of tissue available necessitates prolonged cell expansion. There is therefore interest in alternative sources of adult stem cells for cell-based tissue engineering approaches for cartilage repair. Cells with stem cell characteristics have been reported in many tissues and more recently in subcutaneous adipose tissue and the infrapatellar fat pad (IPFP) [3-6]. Conditions for the differentiation of these cells into chondrocytes, osteoblasts and adipocytes have been used to show that they are multipotent [7]. Because of their multipotency and practical access, cells from the IPFP are of interest as a potential source of cells for the repair of focal cartilage defects in the knee [5]. In previous work we demonstrated the ability of IPFP-derived cells to undergo chondrogenic [8], osteogenic [9] and adipogenic differentiation (W.S. Khan and T.E. Hardingham, unpublished data).

Mammalian cells are normally cultured in air (containing 20% oxygen) with 5% carbon dioxide added, but some cells, including adult stem cells, have been reported to proliferate more rapidly at lower oxygen concentrations [10-12]. Articular cartilage is avascular and exists at a decreased oxygen tension of (1 to 7%) *in vivo *[13,14]. In chondrocyte culture systems it has been shown that under hypoxia there is increased synthesis of extracellular matrix by chondrocytes [15,16], and this has been extended to stem cells from bone marrow [17] and liposuction-derived adipose tissue [14] undergoing chondrogenesis. Thus, oxygen tension seems to be an important regulatory factor in the proliferation, differentiation and matrix production of chondrocytes, but few studies have characterised gene expression changes. In our investigation of the potential of IPFP-derived stem cells from elderly patients undergoing joint replacement for osteoarthritis, we investigated the gene expression changes that characterised the response of these stem cells to hypoxic conditions in chondrogenic cultures.

## Materials and methods

### Cell isolation and culture

The IPFP was obtained with ethical approval and fully informed consent from patients (aged 67, 69 and 72 years; *n *= 3) undergoing total knee replacement for osteoarthritis. The tissue was dissected and cells were isolated by digestion with 0.2% (v/v) collagenase I (Invitrogen, Paisley, Renfrewshire, UK) for 3 hours at 37°C with constant agitation. The released cells were sieved (70 μm mesh) and washed in basic medium, namely DMEM supplemented with 20% (v/v) FCS, 100 U/ml penicillin and 100 μg/ml streptomycin (all from Cambrex, Wokingham, UK), with l-glutamine (2 mM). The stromal cells were separated from the adipocytes (floating) by centrifugation at 300 *g *for 5 minutes and were counted and plated at 100,000 cells/cm^2 ^in monolayer culture in basic medium. Cultures were maintained at 37°C with 5% CO_2 _and normal oxygen (20%). Cultured cells from passage 2 were used for cell surface epitope characterisation and cell aggregate culture.

### Cell surface epitope characterisation and flow cytometry

Confluent passage 2 cells were stained with a panel of antibodies for cell surface epitopes. This included antibodies against the following: CD13 (aminopeptidase N), CD44 (hyaluronan receptor), CD90 (Thy-1), LNGFR (low-affinity nerve growth factor receptor), STRO-1 (marker for bone marrow-derived stem cells) and CD56 (neural cell adhesion molecule; NCAM) from BD Biosciences (Oxford, UK); CD29 (β1 integrin), CD105 (SH2 or endoglin) and CD34 (marker for haematopoetic cells) from Dako (Ely, UK); and 3G5 (marker for vascular pericytes) courtesy of Dr Ann Canfield (University of Manchester, UK). The cells were incubated for 1 hour with the primary mouse antibodies (undiluted 3G5 and 1:100 dilution for others) followed by fluorescein isothiocyanate (FITC)-conjugated anti-mouse IgM secondary antibody (1:40 dilution; Dako). For controls, nonspecific monoclonal mouse IgG antibody (Santa Cruz Biotechnology, Santa Cruz, CA, USA) was substituted for the primary antibody. The cells were incubated with 4',6-diamidino-2-phenylindole stain (1:100 dilution) for 5 minutes, and images were captured with an Axioplan 2 microscope using an Axiocam HRc camera and AxioVision 4.3 software (all from Carl Zeiss Ltd, Welwyn Garden City, UK). Cells from passage 2 were also analysed by flow cytometry. Cells in monolayer were detached with trypsin (0.05%, with 5 mM EDTA), washed and incubated with primary mouse antibodies (undiluted 3G5 and 1:100 dilution for others) followed by FITC-conjugated anti-mouse IgM secondary antibody (1:40 dilution). The cells were washed again, suspended at 10^6 ^cells/ml and assayed in a flow cytometer (Dako cytomation cyan, Ely, UK).

### Cell aggregate culture

Three-dimensional cell aggregates (500,000 cells [18]) were cultured at 37°C in 1 ml of chondrogenic media for 14 days (medium changed every 2 days) in either normal oxygen (95% air containing 20% oxygen, and 5% carbon dioxide) or low oxygen (90% nitrogen, 5% carbon dioxide and 5% oxygen). The chondrogenic culture medium contained basic medium (as above, but without serum) with 1 × insulin–transferrin–selenium supplement (ITS+1; final concentrations 10 μg/ml bovine insulin, 5.5 μg/ml transferrin, 5 ng/ml sodium selenite, 4.7 μg/ml linoleic acid and 0.5 mg/ml BSA), 37.5 μg/ml ascorbate 2-phosphate, 100 nM dexamethasone, 10 ng/ml transforming growth factor (TGF)-β3 and 100 ng/ml insulin-like growth factor-1 (all from Sigma, Poole, UK).

### DNA and glycosaminoglycan assays

The wet mass of cell aggregates was recorded at 14 days and the aggregates were digested overnight at 60°C in 20 μl of 10 U/ml papain (Sigma), 0.1 M sodium acetate, 2.4 mM EDTA, 5 mM l-cysteine pH 5.8. DNA in the papain digest was measured with PicoGreen (Invitrogen) with standard double-stranded DNA (Invitrogen), and sulphated glycosoaminoglycan (GAG) was assayed with 1,9-dimethylmethylene blue (Aldrich, Poole, UK) with shark chondroitin sulphate (Sigma) as standard [18,19].

### Gene expression analysis

Quantitative real-time gene expression analysis was performed for the following: hypoxia-inducible transcription factor (HIF)1α, HIF2α, aggrecan, versican, perlecan, collagen type I (COL1A2), collagen type II (COL2A1), collagen type IX (COL9A1), collagen type X (COL10A1), collagen type XI (COL11A2), L-SOX5, SOX6 and SOX9. Total RNA was extracted with Tri Reagent (Sigma) from passage 2 cells in monolayer and from cell aggregates at 14 days that had been ground with Molecular Grinding Resin (Geno Technology Inc., St Louis, MO, USA). cDNA was generated from 10 to 100 ng of total RNA by using reverse transcription followed by poly(A) PCR global amplification [20]. Globally amplified cDNAs were diluted 1:1,000 and a 1 μl aliquot of the diluted cDNA was amplified by quantitative real-time PCR in a final reaction volume of 25 μl by using an MJ Research Opticon with an SYBR Green Core Kit (Eugentec, Seraing, Belgium). Gene-specific primers were designed within 300 base pairs of the 3' region of the relevant gene with the use of ABI Primer Express software (Applied Biosystems, Foster City, CA, USA). Gene expression analyses were performed relative to β-actin and calculated by using the 2^-ΔΔCt ^method [21]. All primers (Invitrogen) were based on human sequences: aggrecan, 5'-AGGGCGAGTGGAATGATGTT-3' (forward) and 5'-GGTGGCTGTGCCCTTTTTAC-3' (reverse); β-actin, 5'-AAGCCACCCCACTTCTCTCTAA-3' (forward) and 5'-AATGCTATCACCTCCCCTGTGT-3' (reverse); COL1A2, 5'-TTGCCCAAAGTTGTCCTCTTCT-3' (forward) and 5'-AGCTTCTGTGGAACCATGGAA-3' (reverse); COL2A1, 5'-CTGCAAAATAAAATCTCGGTGTTCT-3' (forward) and 5'-GGGCATTTGACTCACACCAGT-3' (reverse); COL9A1, 5'-CGGTTTGCCAGGAGCTATAGG-3' (forward) and 5'-TCTCGGCCATTTTTCCCATA-3' (reverse); COL10A1, 5'-TACCTTGTGCCTCCCATTCAA-3' (forward) and 5'-TACAGTACAGTGCATAAATAAATAATATATCTCCA-3' (reverse); COL11A2, 5'-CCTGAGCCACTGAGTATGTTCATT-3' (forward) and 5'-TTGCAGGATCAGGGAAAGTGA-3' (reverse); HIF1α, 5'-GTAGTTGTGGAAGTTTATGCTAATATTGTGT-3' (forward) and 5'-TCTTGTTTACAGTCTGCTCAAAATATCTT-3' (reverse); HIF2α, 5'-GGTGGCAGAACTTGAAGGGTTA-3' (forward) and 5'-GGGCAACACACACAGGAAATC-3' (reverse); L-SOX5, 5'-GAATGTGATGGGACTGCTTATGTAGA-3' (forward) and 5'-GCATTTATTTGTACAGGCCCTACAA-3' (reverse); SOX6, 5'-CACCAGATATCGACAGAGTGGTCTT-3' (forward) and 5'-CAGGGTTAAAGGCAAAGGGATAA-3' (reverse); SOX9, 5'-CTTTGGTTTGTGTTCGTGTTTTG-3' (forward) and 5'-AGAGAAAGAAAAAGGGAAAGGTAAGTTT-3' (reverse); versican, 5'-TGCTAAAGGCTGCGAATGG-3' (forward) and 5'-AAAAAGGAATGCAGCAAAGAAGA-3' (reverse).

### Immunohistochemical staining of cell aggregate sections

The cell aggregates were fixed for 2 hours in 4% formaldehyde (BDH Ltd, Poole, UK)/Dulbecco's phosphate-buffered solution (DPBS; Cambrex). The samples were then washed in 70% industrial methylated spirit (BDH) and placed in a Shandon Citadel 2000 tissue processor (Thermo Electron Corporation, Runcorn, UK). Paraffin-embedded sections (5 μm) were taken and mounted on slides precoated with Superfrost Plus (Menzel Glaser GmbH, Braunschweig, Germany), dried in air and left at 37°C overnight. Sections were preincubated at 37°C with 0.1 U/ml chondroitinase ABC (Sigma) for 1 hour and then immunostained for 16 hours at 4°C with goat anti-human collagen type I (C-18 polyclonal), or collagen type II (N-19 polyclonal) (both from Santa Cruz Biotechnology) or with rabbit anti-human aggrecan (BR1) (all at 1:100 dilution) followed by washing and incubation for 30 minutes at room temperature in donkey anti-goat IgG for collagen type I and collagen type II and donkey anti-rabbit IgG for aggrecan (all at 1:250 dilution) biotin-conjugated secondary antibodies (both from Santa Cruz Biotechnology). Goat IgG antibody was used as a control for collagen, and rabbit IgG was used as a control for aggrecan (both from Santa Cruz Biotechnology). Endogenous peroxidase activity was quenched for 5 minutes with 3% hydrogen peroxide (Sigma) in methanol (BDH). Non-specific binding was blocked for 1 hour with 10% normal donkey serum diluted in 1% BSA (both from Sigma) in DPBS at room temperature. For visualisation, sections were incubated for 30 minutes at room temperature in streptavidin–peroxidase complex (1:500 in DPBS; Dako), rinsed in distilled water and incubated in fast-DAB (3,3'-diaminobenzidine) peroxidase substrate (Sigma) for 5 minutes and counterstained in diluted filtered haematoxylin (Sigma) for 15 seconds. Images were then taken with an Axioplan 2 microscope with the use of an Axiocam HRc camera and AxioVision 4.3 software.

### Statistical analysis

Experiments were performed separately with cells from three patients, and all experiments were in triplicate. Gene expression data, wet masses, and DNA and GAG assay results are presented as means and SEM. Student's paired *t*-test and a one-way analysis of variance followed by Bonferroni's correction were used to analyse the results from two and three culture conditions, respectively, and to determine the level of significance. Statistical analyses were conducted with SPSS Statistical Software (version 11.5). Significance was set at *p *< 0.05.

## Results

### Isolation, culture and cell surface epitope characterisation of IPFP cells

**Figure 1:**
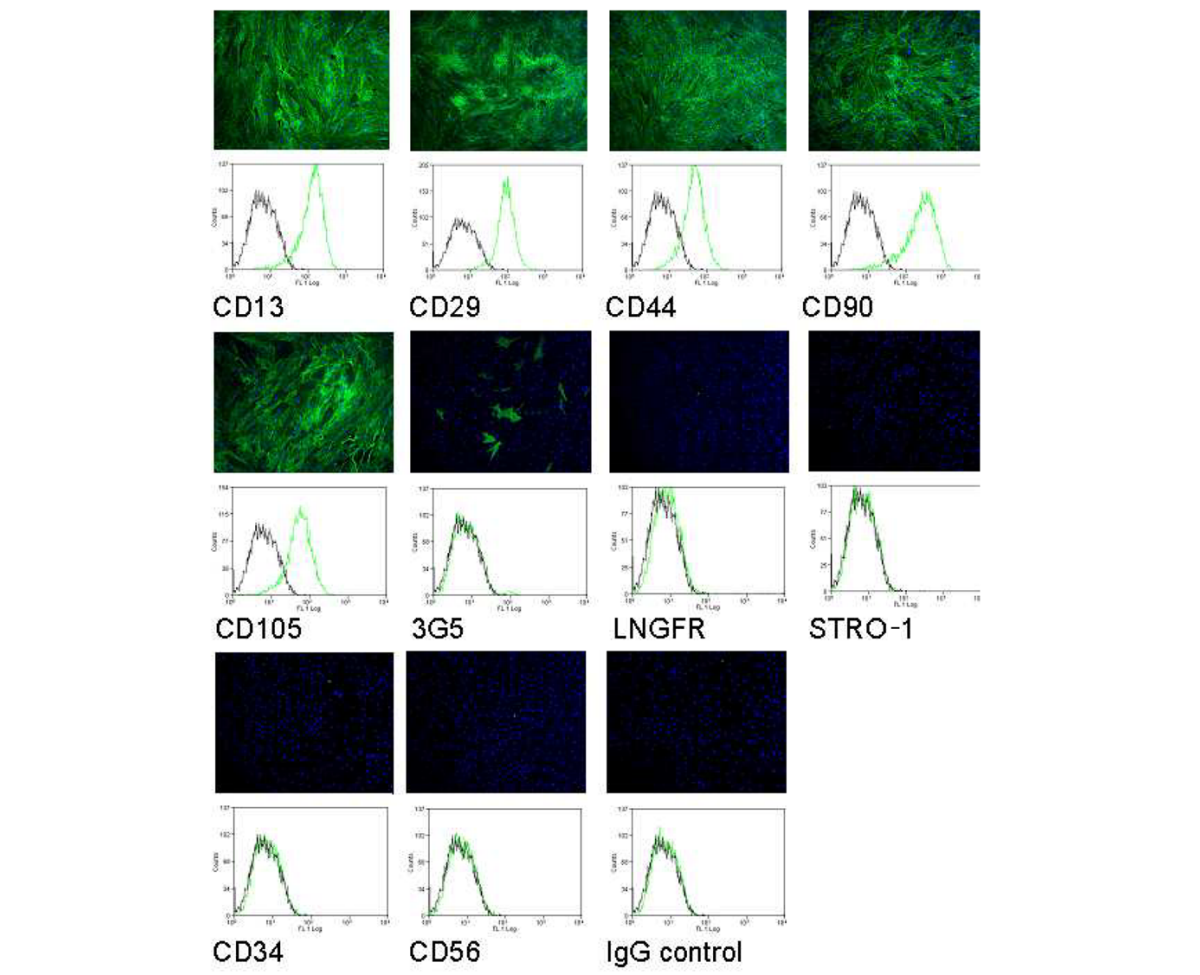
Cell surface epitope characterisation of infrapatellar fat pad cells. Cell surface staining on passage 2 infrapatellar fat pad cells was performed with a panel of antibodies and fluorescein isothiocyanate-conjugated secondary antibody (green), and 4',6-diamidino-2-phenylindole (blue). Results showed strong staining for CD13, CD29, CD44, CD90 and CD105, weak staining for 3G5, and negative staining for low-affinity nerve growth factor receptor (LNGFR), STRO-1, CD34 and CD56. No staining was observed for the IgG control. The staining pattern was confirmed by flow cytometry characterisation and showed the increase in fluorescence (green) compared with the autofluorescence (black).

The cells isolated from the IPFP proliferated in culture and reached confluence by day 14. The yield of cells by the end of passage 2 was typically 10^7 ^cells at confluence from 5 g of IPFP tissue (proliferation rate 0.14 ± 0.01 doublings per day (mean ± SEM) [8]). Cells at passage 2 stained strongly for CD13, CD44, CD90 and CD105 (markers for mesenchymal stem cells), and for CD29 (β1 integrin). The cells stained poorly for LNGFR and STRO-1 (markers on freshly isolated bone marrow stem cells) and sparsely for 3G5 (marker for vascular pericytes). Staining for CD34 (marker for haematopoetic cells) and CD56 (NCAM) was negative. Flow cytometry confirmed the staining pattern and showed the IPFP cell population to be fairly homogeneous for mesenchymal stem cell markers (Figure 1).

### Chondrogenic culture of IPFP cells and the effect of low oxygen tension

**Figure 2:**
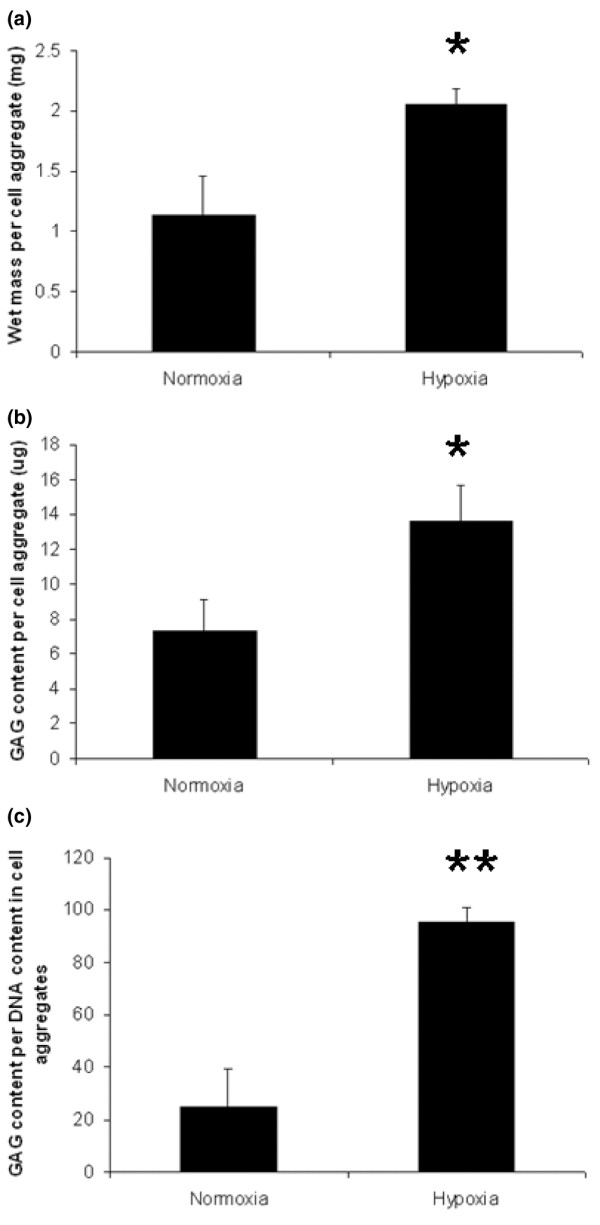
Chondrogenic cultures of infrapatellar fat pad cells and the effects of hypoxia. Wet weight **(a)**, glycosoaminoglycan (GAG) analysis **(b) **and GAG per DNA measurement **(c) **of cell aggregates after chondrogenic differentiation for 14 days under normoxic and hypoxic conditions. Results are means ± SEM (*n *= 3). **p < 0.001; *p < 0.05 (Student's paired *t*-test).

**Figure 3:**
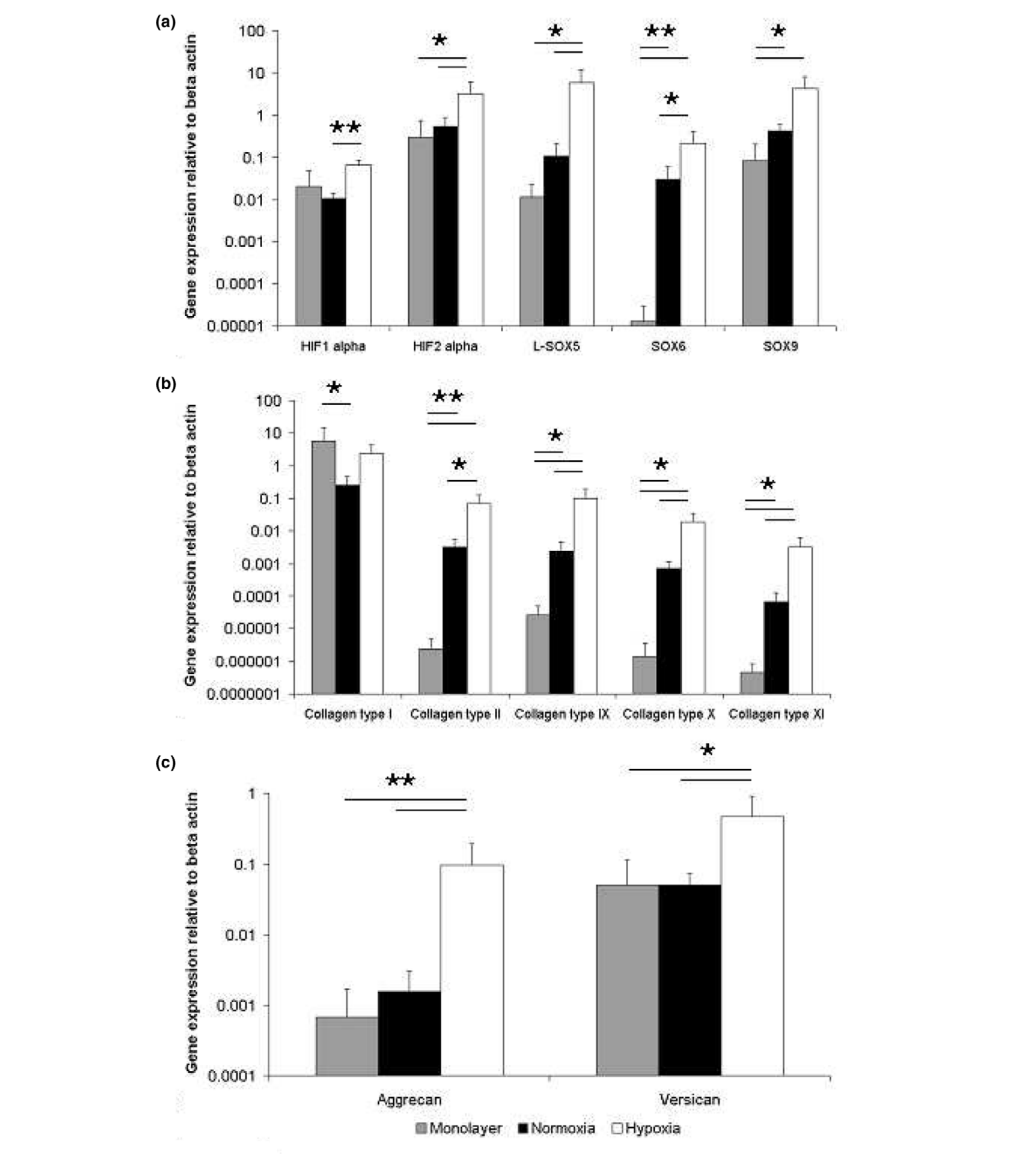
Gene expression in chondrogenic cultures of infrapatellar fat pad cells. Relative gene expression for hypoxia-inducible transcription factors (HIF) and SOX genes **(a)**, collagens **(b) **and proteoglycans **(c) **in monolayer culture and after chondrogenic differentiation for 14 days under normoxic and hypoxic conditions. Results are means ± SEM (*n *= 3). **p < 0.001; *p < 0.05 (analysis of variance with Bonferroni's correction).

The cultured cell aggregates of IPFP cells with chondrogenic medium showed evidence of induction of chondrogenesis under normal culture conditions (20% oxygen), and this was greatly enhanced at a lower oxygen tension (5%) (Figure 2). Cell aggregates cultured under hypoxic conditions at 14 days had 1.8-fold higher wet mass than those cultured under normoxic conditions. The hypoxic conditions resulted in less cell proliferation because the aggregates contained 54% less total DNA. There was, however, a large increase (1.9-fold) in the GAG accumulation (Figure 3) such that the proteoglycan content per cell at 14 days was much higher under hypoxic conditions (3.5-fold, *p *< 0.001; Figure 2).

### Gene expression analysis of chondrogenic IPFP cell aggregates

In the chondrogenic cultures in normal oxygen the gene expression of collagen types II, IX, X and XI and the transcription factors SOX6 and SOX9 was greatly increased (*p *< 0.05 or *p *< 0.001) in comparison with monolayer culture. In contrast, the expression of the proteoglycans, aggrecan and versican did not change (Figure 3). In the presence of lowered oxygen tension there was a more enhanced chondrogenic response with additional changes in gene expression (Figure 3). In low oxygen the expression of HIF2α was increased 11-fold over monolayer culture (Figure 3); interestingly, there was no change in HIF1α, which was expressed at a lower level than HIF2α in monolayer. The expression of collagen types II, IX, X and XI at lowered oxygen tension was increased 30,000-fold, 4,000-fold, 14,000-fold and 7,000-fold, respectively, over monolayer culture (*p *< 0.05 or *p *< 0.001), and SOX5, SOX6 and SOX9 were increased 500-fold, 17,000-fold and 50-fold, respectively (*p *< 0.05 or *p *< 0.001; Figure 3). In addition, aggrecan was greatly increased (140-fold) and versican was increased slightly less (9-fold); both were unchanged in normoxia. Collagen type I (COL1A2) was highly expressed in monolayer-cultured IPFP cells; this remained high in the chondrogenic cultures and there was no downregulation of its expression under hypoxic conditions. The chondrogenic response of IPFP cells in cell aggregate culture was thus greatly enhanced over 14 days at lowered oxygen tension, and this was correlated with the selective upregulation of HIF2α and increased expression of the key chondrogenic transcription factors SOX9, SOX5 and SOX6.

### Immunohistochemistry of chondrogenic IPFP cell aggregates

**Figure 4:**
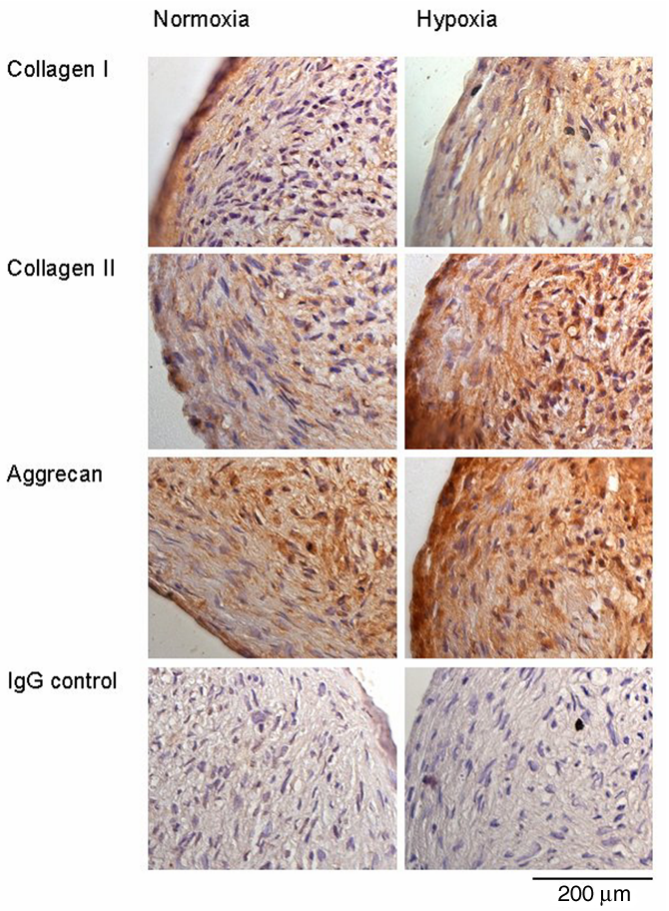
Immunohistochemistry of chondrogenic cultures of infrapatellar fat pad cells. Immunohistochemical staining for collagen type I and II, aggrecan and control IgG in cell aggregates after chondrogenic differentiation for 14 days under normoxic and hypoxic culture conditions.

The cell aggregates cultured under both normoxic and hypoxic conditions showed evidence of chondrogenesis with immunolocalisation of cartilage-associated matrix, including collagen type II and aggrecan (Figure 4). Cell aggregates cultured under hypoxic conditions were larger and less cellular than aggregates cultured under normoxia. All cells had a rounded appearance and were surrounded by extracellular matrix. Cell aggregates under normoxic and hypoxic conditions both stained, albeit weakly, for collagen type I, although the hypoxic cultures lacked the collagen I peripheral rim seen in normoxia, suggesting a lower level of collagen I production.

## Discussion

The cell surface epitope characterisation and flow cytometry of the IPFP cell population showed a similar staining pattern to that of bone marrow-derived stem cells, and although they stained poorly for STRO-1 and for LNGFR, the expression of these markers on bone marrow-derived stem cells is reported to decline with culture [22-25]. In preliminary work the cells have shown osteogenic [9] and adipogenic (W.S. Khan and T.E. Hardingham, unpublished data) differentiation. It has also previously been noted that other adipose tissue-derived stem cells did not express STRO-1 [26]. In IPFP tissue sections we have identified perivascular cells, which stained with the antibody 3G5 [27]. The antigen recognised by 3G5 is a cell surface ganglioside, characterised originally on vascular pericytes from bovine retina, which have been shown to have multidifferentiation potential [28-32]. In the cultured IPFP cells only 3 to 7% stained positively for 3G5, and the fraction that stained did not change with further passage; we have also observed that with clonally expanded IPFP cells only a small proportion of the progeny of each stained with 3G5 (W.S. Khan and T.E. Hardingham, unpublished data), suggesting this was not a separate subpopulation. These results suggested that the culture conditions did not favour 3G5 expression, and previously it has been reported to vary in culture [33]. It may therefore be possible that the IPFP cells isolated were derived from those staining with 3G5 in the tissue, but that in culture 3G5 was expressed on only few cells at any one time.

The IPFP cells responded to chondrogenic culture in cell aggregates, and this was much enhanced under hypoxic conditions. The wet mass of cell aggregate provides a simple measure of *in vitro *chondrogenesis in mesenchymal stem cells [34,35]; cell aggregates cultured under hypoxic conditions had a 1.8-fold higher wet mass than those cultured under normoxia. The GAG content reflected proteoglycan biosynthesis and accumulation in the matrix, and under hypoxic conditions there was a 1.9-fold increase in the total GAG per aggregate. In spite of the increased mass there was a lower DNA content than under normoxia, reflecting a lower cell proliferation rate, and this was balanced by a much greater production of GAG per cell. These results with IPFP cells are comparable to those from a previous study on stem cells derived from other human liposuction-derived adipose tissue, in which 5% oxygen was reported to increase collagen and GAG synthesis and reduce cell proliferation [14]. However, with mouse inguinal fat-derived cells, decreased chondrogenesis and osteogenesis was reported in 2% oxygen [36]. The effects of hypoxia on the propagation of human bone marrow-derived stem cells suggested that 2% oxygen favoured more primitive stem cells with higher colony-forming unit capability and stronger expression of stem cell genes, and that when switched to normoxic conditions they showed stronger osteoblastic and adipocytic differentiation [37].

The gene expression analysis provided an assessment of the changes induced by hypoxia in the chondrogenic cultures, and the gene expression in monolayer culture provided a measure of expression before the cell aggregates were formed. The transcription factor SOX9 has been shown to be essential for chondrocyte differentiation and cartilage formation [38]. One of its actions is to activate specific enhancer elements in cartilage matrix genes such as collagen type II and aggrecan [39,40]. This action of SOX9 is further enhanced by SOX5 and SOX6. In the chondrogenic cultures of IPFP cells there was an increase in the expression of SOX9, which was already expressed at a significant level and this was increased more strongly in hypoxia. SOX5 and SOX6 showed different proportionate responses, as SOX5 was not increased under normoxia but was increased by hypoxia, whereas SOX6 was upregulated in normoxia and this was further enhanced by hypoxia. The net effect was that under hypoxia there was higher expression of SOX5, 6 and 9 than under normoxic conditions.

The expression of key cartilage collagens II, IX and XI were all correspondingly increased under hypoxia, showing that hypoxia enhanced the potential for the assembly of a complete cartilage fibrillar template. Although TGF-β inhibits the terminal differentiation of chondrocytes *in vivo *[41] there was also a higher level of collagen type X expression in IPFP chondrogenic cultures. However, TGF-β has previously been associated with increased expression of collagen type X in chondrogenesis in bone marrow stem cells [42]. Aggrecan, and to a smaller extent versican, were also increased in hypoxia.

The response by cells to hypoxia is complex and is mediated by several genes [43]. HIF1α is one of the major regulators of hypoxic response in most cells and tissues [44], where it is frequently associated with angiogenesis and the formation of new blood vessels. Targets of its molecular signalling are reported to include a cluster of hydroxylases that are crucial for collagen fibre formation such as prolyl 4-hydroxylase and procollagen lysyl-hydroxylase [45-47]. Through these actions, HIF1α affects the rate of synthesis of procollagen chains *in vivo *and *in vitro *[48]. HIF2α is closely related to HIF1α, with similarities in DNA binding and dimerisation, but with differences in transactivation domains [49]. The genes downstream of HIF2α have been less well characterised, but it seems to act through some of the pathways common to HIF1α. Both genes are upregulated together in some cells, but many examples of selective activation of one or the other are known, and an increasing number of cell-type-specific gene targets have been identified [49,50]. It was therefore an important finding here that HIF2α and not HIF1α was upregulated in the IPFP cells in response to 5% oxygen. It suggests that in chondrogenic IPFP cells the response to hypoxia is mediated by HIF2α and that this helps to drive the increased matrix production, assisted by increased expression of the hydroxylases necessary for collagen fibril formation. It may be an important factor that this was the response in 5% oxygen, which is a low oxygen tension but within the physiological range for chondrocytes [13]. In other chondrogenic systems we have noted reduced matrix production with cell aggregates in 1% oxygen (A.B. Adesida and T.E. Hardingham, unpublished data). It has also been noted that only HIF2α, and not HIF1α, was upregulated in neuroblastoma cells cultured in 5% oxygen, whereas HIF1α and HIF2α were both upregulated when the oxygen tension was decreased to 1% [51]. In the context of these cultures the oxygen concentration is 5% at the surface but is likely to be below 5% towards the centre of the aggregate. It has also been noted that in prolonged hypoxia in lung epithelial cells, the upregulation of HIF1α was transient, whereas increases in HIF2α were sustained [52]. A transient upregulation of HIF1α cannot be ruled out in these cultures, but the expression of HIF1α was much lower than HIF2α under all conditions (Figure 3) and it is clear that at 14 days under hypoxic conditions HIF2α was expressed about 40-fold more than HIF1α. The present results suggest that HIF2α expression in chondrogenic cells may act to selectively enhance the expression of the cartilage matrix genes. However, this may be an indirect action through the increased expression of the transcription factors SOX9, SOX5 and SOX6. The differential effect of hypoxia on the expression of the cartilage matrix genes suggests that the cartilage collagens may become actively expressed at lower levels of SOX9, SOX5 and SOX6 transcription factors, whereas aggrecan may require higher levels of SOX9, SOX5 and SOX6 to achieve full expression.

## Conclusion

Our results show that cells with stem cell or progenitor cell characteristics can be isolated from the IPFP derived from elderly patients with osteoarthritis. Cells from each patient tested (*n *= 3) showed the ability to undergo chondrogenic differentiation, and this was enhanced in 5% oxygen. This is the first study that has characterised chondrogenic gene expression in IPFP-derived cells. Our results extend previous observations and identify here most importantly that HIF2α, and not HIF1α, was upregulated in response to lowered oxygen tension in the chondrogenic cultures. The results showed that chondrogenesis was enhanced in an atmosphere of decreased oxygen tension and that this is mediated by a significantly increased expression of key genes expressed by chondrocytes, notably the transcription factors SOX5, SOX6 and SOX9. These findings show that oxygen tension has an important role in regulating the synthesis and assembly of matrix by IPFP-derived stem cells as they undergo chondrogenesis and that this has important implications for the use of the IPFP in cartilage tissue engineering.

## Abbreviations

ANOVA = analysis of variance; BSA = bovine serum albumin; DMEM = Dulbecco's modified Eagle's medium; DPBS = Dulbecco's phosphate-buffered solution; FCS = fetal calf serum; FITC = fluorescein isothiocyanate; GAG = glycosoaminoglycan; HIF = hypoxia-inducible transcription factor; IPFP = infrapatellar fat pad; LNGFR = low-affinity nerve growth factor receptor; NCAM = neural cell adhesion molecule; PCR = polymerase chain reaction; TGF = transforming growth factor.

## Competing interests

The authors declare that they have no competing interests.

## Authors' contributions

WSK conceived, designed and performed the experiments described in this study, was responsible for tissue procurement and processing, and produced the initial version of this manuscript. ABA helped conceive the experiments and perform the gene expression analyses. TEH supervised and oversaw the experiments and writing of this manuscript. All authors read and approved the final manuscript.
